# Sequence dependent variations in RNA duplex are related to non-canonical hydrogen bond interactions in dinucleotide steps

**DOI:** 10.1186/1756-0500-7-83

**Published:** 2014-02-07

**Authors:** Senthilkumar Kailasam, Dhananjay Bhattacharyya, Manju Bansal

**Affiliations:** 1 Molecular Biophysics Unit, Indian Institute of Science, Bangalore 560012, India; 2 Computational Science Division, Saha Institute of Nuclear Physics, 1/AF Bidhan Nagar, Kolkata 700064, India

**Keywords:** RNA, Dinucleotide, Hydrogen bond, RNA-Protein, Watson-Crick, Basepairs

## Abstract

**Background:**

Sequence determines the three-dimensional structure of RNAs, and thereby plays an important role in carrying out various biological functions. RNA duplexes containing Watson-Crick (WC) basepairs, interspersed with non-Watson-Crick basepairs, are the dominant structural unit and form the scaffold for the 3-dimensional structure of RNA. It is therefore crucial to understand the geometric variation in the dinucleotide steps that form the helices. We have carried out a detailed analysis of the dinucleotide steps formed by AU and GC Watson-Crick basepairs in RNA structures (both free and protein bound) and compared the results to that seen in DNA. Further, the effect of protein binding on these steps was examined by comparing steps in free RNA structures with protein bound RNA structures.

**Results:**

Characteristic sequence dependent geometries are observed for the RR, RY and YR type of dinucleotide steps in RNA. Their geometric parameters show correlated variations that are different from those observed in B-DNA helices. Subtle, but statistically significant differences are seen in roll, slide and average propeller-twist values, between the dinucleotide steps of free RNA and protein bound RNA structures. Many non-canonical cross-strand and intra-strand hydrogen bonds were identified that can stabilise the RNA dinucleotide steps, among which YR steps show presence of many new unreported interactions.

**Conclusions:**

Our work provides for the first time a detailed analysis of the conformational preferences exhibited by Watson-Crick basepair containing steps in RNA double helices. Overall, the WC dinucleotide steps show considerable conformational variability. Furthermore, we have identified hydrogen bond interactions in several of the dinucleotide steps that could play a role in determining the preferred geometry, in addition to the intra-basepair hydrogen bonds and stacking interactions. Protein binding affects the conformation of the steps that are in direct contact, as well as allosterically affect the steps that are not in direct physical contact.

## Background

The double helical structure of nucleic acids exists in various polymorphic sub-states, of which DNA prefers B-form conformation and RNA prefers A-form. The A-form in RNA is a right-handed helix formed by stacking of Watson-Crick (WC) basepairs along with a few non-Watson-Crick (NWC) basepairs. The overall conformation of the helices is dictated by the geometry of successive dinucleotide steps, which in-turn is dictated by the chemical nature of bases involved in forming these step. A-form helix is characterized by large roll angle and negative slide values, compared to B-form and is accompanied by a narrow but deep major groove and a wide, shallow minor groove [1]. Significant progress has been made in the understanding of sequence dependent conformational preference of dinucleotide steps in DNA [2-6], while the geometric preference in RNA helices remains largely unexplored. Information on the geometric preference at the step level, can contribute to the overall understanding of the structural organization of RNA. A database of all possible dinucleotide steps with their step parameter values is available in the public domain [7], but there is very little explicit discussion about the conformational features of various dinucleotide steps observed in RNA.

The helical regions in DNA are comprised almost exclusively of canonical Watson-Crick (AT and GC) basepairs, which form 10 unique dinucleotide steps. On the contrary, an RNA duplex consists of canonical WC basepairs (AU and GC), that are paired along Watson-Crick edge in *cis* orientation (cisWW family), as well as basepairs involving the Hoogsteen and Sugar edges. In addition, other non-canonical basepairs from the cisWW family e.g. GU, GA, UU also occur frequently in the RNA duplex [8-11]. Thus, the type of dinucleotide steps that can occur in RNA is much larger and complicates any analysis of their sequence dependent conformations. In the present work, we focus only on the dinucleotide steps formed by the two canonical WC basepairs (AU and GC) of the cisWW family, which constitute a major proportion of the helical steps and compare them to the corresponding A-like and B-like DNA steps.

The diverse RNA structural motifs and equally diverse proteins that interact with RNA suggest that the conformational changes that occur during RNA-protein interaction can be characterized by changes in the protein, the RNA, or both [12]. An analysis of crystal structures of free and RNA bound proteins, suggested that the proteins do not show any significant change on binding to RNA [13]. A number of studies on RNA-protein interaction have focused on the RNA-protein interface and recognition mechanism [14-16], but none have analyzed the protein induced conformational change, if any, in RNA. On the other hand, the effect of protein binding on dinucleotide steps of DNA is well documented [4,17-19]. Specific RNA-binding domains recognize the sequence and shape of the interacting region [20], while others interact in a non-specific manner. The wide and shallow minor groove present in RNA helix allows easy access for interaction with protein. However, sometimes, the narrow and deep major groove can also interact with proteins owing to presence of mismatch basepairs and bulges along the helix that lead to widening of the major groove [21]. Understanding the conformational changes induced by the interaction of protein, on the dinucleotide steps of the RNA helix, can help deduce general mechanism involved in RNA-protein recognition.

The geometry of dinucleotide step is mainly influenced by basepair hydrogen bonds and stacking interactions [22-24]. Apart from the standard hydrogen bonds involved in basepairing, additional interactions involving base, ribose sugar (especially O2′ group) and phosphate atoms in the RNA backbone were reported in RNA structures [25-27]. These interactions mainly occur in hairpin, internal or junction loops or as part of tertiary interactions. In the light of the developments in our understanding of ‘weak hydrogen bonds’ [28,29], the importance of such interactions in the structure formation, folding and stability of various macromolecules are also being investigated [30-33]. Presence of potentially weak cross-strand and intra-strand hydrogen bond interactions in dinucleotide steps of B-DNA crystal structures have been reported and analyzed [30,34,35]. In RNA crystal structures, such interactions between bases in a dinucleotide step have not been reported. Moreover, the characteristic A-like geometry seen in RNA helices can possibly prevent their formation or favor some novel interactions. Recent molecular dynamic (MD) simulation studies on modeled RNA duplexes suggest that potential interactions in a dinucleotide steps are present between exo-cyclic atoms [36]. Hence, identification of these additional stabilizing forces in various dinucleotide steps in RNA crystal structures can help understand their preferred geometry and in *ab initio* modeling of RNA structures.

In this work, a non-redundant RNA crystal structure dataset has been created and we have examined the intrinsic geometries of all WC basepairs and the dinucleotide steps formed by them in the helical regions. To understand the extent of conformational variability that can occur in the dinucleotide steps, the effect of protein binding on these steps was examined by comparing the helices in free RNA with protein bound RNA dataset. Further, we have carried out a systematic analysis of dinucleotide steps to identify potential hydrogen bond interactions between all four bases and correlated the occurrence of such bonds with the dinucleotide step geometry.

## Methods

### Preparation of dinucleotide dataset

The x-ray crystal structure dataset was created by extracting structures with resolution better than 3.0 Å from the Protein Data Bank [37]. The dataset was made non-redundant using the web servers HD-RNAS [38] and FR3D [39]. Non-standard bases and other chemically modified bases were not included in this study. RNA structures that are not bound to proteins were grouped as *‘free-RNA’* dataset and those that were in complex with a protein were grouped as *‘bound-RNA’* dataset (Table 1). A non-redundant free DNA dataset containing structures with resolution better than 2.0 Å was created to compare it with RNA datasets. The 10 dinucleotide steps in DNA helices were grouped into A-like and B-like steps, based on their Zp value [4]. They are referred to as *‘ADNA’* and *‘BDNA’* dataset respectively. The parameters obtained from crystal datasets are also compared with those of fibre diffraction models. A standard B-DNA fibre model (*fibre-BDNA*) was generated using NUCGEN [40]. Unlike RNA crystal structures, the basepairs in RNA fibre models in the literature have small negative propeller-twist (-2.1°). 3DNA v2.1 has the option to generate a uniform A-RNA double helix with large negative propeller-twist (-10.5°) [41,42]. We have used this RNA model (referred henceforth as ‘*ModelRNA*’) in our analysis for a more realistic comparison with crystal RNA datasets.

**Table 1:** **List of PDB IDs of structures in *free-RNA, bound-RNA *** **and DNA dataset**

**Dataset (n)**	**PDB ID**
*free-RNA *(88)	157D, 1CSL, 1DQH, 1DUQ, 1EHZ, 1EVV, 1 F27, 1GID, 1HR2, 1I9X, 1KFO, 1KH6, 1L2X, 1LC4, 1MHK, 1NLC, 1NTB, 1NUJ, 1NYI, 1Q29, 1QC0, 1RNA, 1SDR, 1T0D, 1T0E, 1U8D, 1U9S, 1X9C, 1XJR, 1Y26, 1Y27, 1YFG, 1YZD, 1Z79, 1Z7F, 1ZCI, 1ZEV, 1ZFT, 1ZFV, 1ZFX, 1ZX7, 205D, 255D, 280D, 283D, 2A43, 2AO5, 2B57, 2D2K, 2D2L, 2ET5, 2FGP, 2FQN, 2G92, 2H1M, 2OE5, 2OE8, 2OEU, 2OIY, 2PN4, 2PWT, 2Q1O, 2Q1R, 2R20, 2Z75, 353D, 354D, 361D, 364D, 397D, 3B31, 3B4B, 3B5S, 3CJZ, 3CZW, 3D0X, 3D2V, 3DIL, 3DS7, 3FS0, 3FTM, 3GCA, 3GER, 406D, 413D, 420D, 430D, 433D
*bound-RNA *(127)	1A9N, 1B23, 1DFU, 1DI2, 1E7K, 1EC6, 1EFW, 1F7V, 1F7Y, 1FEU, 1G1X, 1GAX, 1H3E, 1H4S, 1I6U, 1IL2, 1J1U, 1JID, 1K8W, 1LNG, 1M5O, 1MJI, 1MZP, 1 N35, 1OOA, 1Q2R, 1QA6, 1QF6, 1QRS, 1QU2, 1R3E, 1R9F, 1RPU, 1S03, 1S72, 1SER, 1TFW, 1U0B, 1URN, 1VFG, 1ZBH, 2ANN, 2AZ2, 2AZX, 2B3J, 2BGG, 2BH2, 2BTE, 2CSX, 2CV1, 2DLC, 2DR8, 2DU3, 2E9T, 2F8K, 2F8S, 2FMT, 2HW8, 2I82, 2NUG, 2NZ4, 2OZB, 2PJP, 2PXV, 2QUX, 2RFK, 2VPL, 2XD0, 2Y8Y, 2ZI0, 2ZJR, 2ZM5, 2ZZM, 3A6P, 3ADD, 3AKZ, 3 AM1, 3AMT, 3AVX, 3BSO, 3CUN, 3DH3, 3EGZ, 3EPH, 3EQT, 3FTF, 3HAX, 3HHN, 3HJW, 3IAB, 3KFU, 3KMQ, 3KS8, 3 L25, 3LRR, 3MOJ, 3MQK, 3NCU, 3NVI, 3OIN, 3OL8, 3OVA, 3QRP, 3R2D, 3R9X, 3RW6, 3SIU, 3SNP, 3TMI, 3TS2, 3UCZ, 3UMY, 3V2F, 3V7E, 3VJR, 4AL5, 4AQ7, 4ATO, 4AY2, 4ERD, 4FVU, 4GCW, 4GD2, 4GHA, 4GHL, 4HXH, 4IG8,
DNA (76)	118D, 126D, 137D, 138D, 158D, 160D, 196D, 1D13, 1D23, 1D49, 1D56, 1D57, 1D79, 1D8G, 1 DC0, 1DNZ, 1DOU, 1EHV, 1EN3, 1EN9, 1ENN, 1IKK, 1 M77, 1P4Z, 1S23, 1SGS, 1SK5, 1VJ4, 1WQY, 1XJX, 1XJY, 1ZEX, 1ZEY, 1ZF0, 1ZF1, 1ZF5, 1ZF6, 1ZF7, 1ZF8, 1ZF9, 1ZFA, 1ZFB, 1ZFC, 1ZFF, 1ZFG, 220D, 221D, 240D, 243D, 260D, 2A7E, 2B1B, 2D94, 2D95, 307D, 317D, 348D, 349D, 368D, 369D, 370D, 371D, 395D, 396D, 399D, 414D, 423D, 431D, 441D, 463D, 476D, 477D, 5DNB, 7BNA, 9BNA, 9DNA

The number of PDB structures included in each dataset is given within parenthesis. The steps in DNA duplex were sub-grouped into *ADNA* and *BDNA* based on their Zp value [4].

### Intra-basepair and dinucleotide step parameters

The helical structures in each of the datasets were subjected to geometry based identification and classification of basepairs using BPFind program with default criteria [8]. Helical stems containing 4 or more basepairs alone were included in the datasets. We analyzed steps formed by AU and GC basepair combinations of the cisWW family [43]. The dinucleotide steps were grouped based on their sequence. The six intra-basepair and six dinucleotide step parameters were calculated using NUPARM program [40,44]. All the intra-basepair and dinucleotide step parameters were calculated using the default option of line joining C6 - C8 atoms as y-axis. Additionally, Zp and cup parameters were also calculated for each step. ‘Zp’ relates the basepairs of the step to their backbone geometries [45]. In a dinucleotide step, it gives the displacement of phosphate atoms in each strand from the midplane between the two stacked basepairs and is the best discriminator between A-form and B-form conformation [4,19]. ‘Cup’ is the difference in buckle parameter between the two basepairs that form the dinucleotide step. Correlation between parameters was analysed and their statistically significant difference (P < 0.01) were checked using Pearson- Correlation coefficient (r) value. The distribution of the data was shown using a Mahalanobis ellipse fitted on to datapoints with the mean as centre and to cover 90% of the datapoints in each group. The stacking area overlap between the basepairs forming a dinucleotide step was calculated using 3DNA program [42]. Stacking area overlap was calculated between bases by including all atoms, as well as the ring atoms alone (excluding exo-cyclic atoms). The total stacking area overlap is the sum of two intra-strand and two cross-strand overlap between the 4 bases involved in the step.

### Hydrogen bond analysis

Hydrogen atoms coordinates was added to all the crystal structures using REDUCE program [46]. The following criteria were used to identify hydrogen bonds (i) donor to acceptor distance (D..A) ≤ 3.8 Å, (ii) Angle D - H..A ≥ 90°. Only bonds that are observed in more than 50% of the cases in each of the 10 dinucleotide steps are discussed.

### Dinucleotide steps interacting with protein

In the *bound-RNA* dataset, the interactions between the atoms in a dinucleotide steps and the protein atoms were identified using CONTACT program in CCP4 program suite [47]. A contact distance of ≤ 4 Å between any pair of amino acid and RNA atom was considered to be interacting. Thus, the steps in the bound RNA dataset were sub-classified into two datasets, those that are in contact with protein (*cont*) and those that are not in contact (*non-cont*). In order to assess if the difference in step parameter values between the various datasets was significant, an unpaired student-t-test was carried out.

MATLAB was used for all statistical analysis and for plotting graphs [48].

## Results

The *free-RNA* dataset consists of 88 protein-free x-ray crystal structures, while *bound-RNA* dataset includes 127 structures (Table 1). Canonical WC basepairs (AU and GC) constitute more than 83% of the total basepairs in these structures and ~ 74% of dinucleotide steps are comprised of these basepairs (Table 2). This work focuses on the sequence dependent conformational preferences of the 10 dinucleotide steps formed by WC basepairs and are compared with those observed in A-DNA and B-DNA helices.

**Table 2:** Occurrence and percentage frequency of base pairs and dinucleotide steps present in the various datasets

***Datasets***	***Base pairs***		***Dinucleotide steps***	
	***Total***	***WC pairs***	***Total***	***WC steps***
		***(%)***		***(%)***
*free-RNA*	1254	1064	1060	797
		(84.9)		(75.2)
*bound-RNA*	4113	3338	3510	2531^¥^
		(81.2)		(72.1)
*ADNA*	316	316	195	195
		(100%)		(100%)
*BDNA*	320	320	212	212
		(100%)		(100%)

Total number of base pairs and dinucleotide steps in the helical region in each dataset are listed, along with the number of canonical WC basepairs and steps comprised of only WC basepairs. The percentage occurrence of WC basepairs and steps is given within parenthesis.

^¥^61.9% of the total WC steps (1566) in the *bound-RNA* dataset are in direct contact with protein and comprise the ‘*cont*’ dataset, while remainder constitute the ‘*non-cont*’ dataset.

### Dinucleotide step geometries

The intra-basepair parameters of WC basepairs present in the RNA datasets are comparable to that of the model structure but are characterized by large variations. The only noticeable features are that GC basepairs have higher negative buckle compared to AU basepairs, while AU basepairs show higher open angle value compared to GC (Table 3). Overall, AU and AT basepairs have lower buckle, slightly larger negative propeller-twist and open angle values compared to GC basepairs, in both RNA and *ADNA* crystal structures. RNA helices are GC rich but the datasets contain a good representation of all 10 unique dinucleotide steps. These dinucleotide steps are generally sub-grouped into three broad categories; Purine-Purine (RR)/ Pyrimidine-Pyrimidine (YY), Purine-Pyrimidine (RY) and Pyrimidine-Purine (YR). The mean and standard deviation values of the step parameters, along with the corresponding average propeller-twist, cup and Zp values, for the three types of dinucleotide steps in the *free-RNA* and *bound-RNA* dataset are tabulated in Tables 4, 5 and 6. The parameter values for the *ModelRNA* are also listed. A comparison of the crystal structure geometries with the model structure values indicates that these steps show some characteristic sequence dependent preferences in their geometries.

**Table 3:** Intra-basepair parameters for W-C basepairs

***Base Pair (n)***	***Buckle***	***Propeller-twist***	***Open***	***Shear***	***Stretch***	***Stagger***	***λ1***	***λ2***	***C1'-C1'***	***C6-C8***
***ModelRNA***										
AU	-1.5	-10.5	-0.2	0.0	2.9	-0.1	54.4	54.4	10.7	9.9
GC	-1.3	-10.5	-1.3	0.0	2.9	-0.1	54.4	54.4	10.7	9.9
***free-RNA***										
AU	-1.5	-12.7	3.6	0.1	2.8	0.0	56.0	55.8	10.5	9.8
(339)	(5.6)	(5.3)	(3.9)	(0.3)	(0.1)	(0.2)	(2.6)	(3.0)	(0.2)	(0.1)
GC	-5.1	-10.9	0.9	-0.1	2.9	0.0	54.2	55.3	10.6	9.9
(725)	(6.5)	(6.0)	(3.3)	(0.3)	(0.1)	(0.2)	(2.4)	(2.9)	(0.2)	(0.1)
***bound-RNA***										
AU	-2.8	-9.5	3.7	0.1	2.8	0.0	56.0	55.6	10.5	9.8
(862)	(8.8)	(7.2)	(4.8)	(0.4)	(0.2)	(0.4)	(3.3)	(3.5)	(0.3)	(0.1)
GC	-6.1	-8.9	0.9	-0.1	2.9	-0.1	54.3	55.1	10.6	9.9
(2476)	(8.8)	(7.0)	(3.9)	(0.4)	(0.1)	(0.3)	(3.1)	(3.2)	(0.2)	(0.2)
***ADNA***										
AT	-0.3	-9.7	2.8	0.0	2.8	-0.1	55.1	56.5	10.4	9.8
(43)	(6.3)	(5.6)	(4.6)	(0.2)	(0.1)	(0.2)	(4.0)	(3.3)	(0.2)	(0.1)
GC	-6.6	-9.4	-0.2	-0.1	2.9	-0.1	55.2	55.1	10.6	9.8
(273)	(7.7)	(6.5)	(2.8)	(0.2)	(0.1)	(0.3)	(2.7)	(2.7)	(0.2)	(0.1)
***BDNA***										
AT	-2.1	-13.7	4.2	0.1	2.8	-0.1	55.5	55.7	10.5	9.8
(136)	(7.8)	(5.3)	(4.1)	(0.3)	(0.1)	(0.2)	(3.2)	(3.2)	(0.2)	(0.1)
GC	0.1	-9.2	-0.7	-0.1	2.9	0.0	54.7	54.5	10.7	9.9
(184)	(9.3)	(7.2)	(3.4)	(0.3)	(0.1)	(0.2)	(2.8)	(2.8)	(0.2)	(0.2)

Mean and standard deviation (SD) values of intra-basepair parameters for all RNA and DNA datasets are tabulated. Frequency of occurrence of each base pair and SD of the parameters are given in parenthesis. Values for Buckle, Open, Propeller-twist, λ1 and λ2 are in degrees (°). Stagger, Shear, Stretch, C1′-C1′ and C6-C8 distances are in Angstroms (Å).

**Table 4:** Dinucleotide step parameters values for RR steps in RNA helices

**Step**	**Dataset ** **(n)**	**Tilt**	**Roll**	**Twist**	**Shift**	**Slide**	**Rise**	**Prop.av**	**Cup**	**Zp**
AA/UU	*free-RNA*	1.1	7.6	32.6	0.1	-1.1	3.3	-14.3	2.3	2.0
	(40)	(1.3)	(3.1)	(2.1)	(0.3)	(0.3)	(0.1)	(3.8)	(6.4)	(0.3)
	*non-cont*	0.9	6.8	30.5	0.1	-1.3	3.3	-10.6	2.0	2.2
	(30)	(3.1)	(5.7)	(2.9)	(0.4)	(0.5)	(0.2)	(6.9)	(11.7)	(0.4)
	*cont*	0.8	6.7	30.7	-0.0	-1.5	3.3	-10.0	4.1	2.2
	(52)	(2.6)	(5.2)	(3.5)	(0.6)	(0.4)	(0.1)	(6.6)	(9.9)	(0.3)
AG/CU	*free-RNA*	0.4	9.7	32.2	0.1	-1.4	3.3	-12.8	-3.8	2.2
	(82)	(2.0)	(3.0)	(2.4)	(0.4)	(0.4)	(0.1)	(3.7)	(6.2)	(0.2)
	*non-cont*	0.6	8.4	31.4	0.2	-1.6	3.3	-10.6	-2.8	2.3
	(107)	(2.7)	(4.2)	(3.3)	(0.5)	(0.4)	(0.1)	(5.3)	(8.6)	(0.3)
	*cont*	1.3	8.0	30.6	0.2	-1.7	3.3	-9.0	-2.2	2.3
	(172)	(2.6)	(4.3)	(3.4)	(0.5)	(0.5)	(0.1)	(5.5)	(10.2)	(0.3)
GA/UC	*free-RNA*	0.2	6.4	31.5	-0.1	-1.5	3.3	-11.2	1.5	2.2
	(68)	(2.0)	(4.1)	(2.3)	(0.4)	(0.4)	(0.1)	(5.5)	(7.1)	(0.3)
	*non-cont*	0.7	6.1	31.1	-0.0	-1.7	3.3	-8.9	1.4	2.4
	(116)	(2.9)	(5.0)	(3.2)	(0.5)	(0.4)	(0.1)	(6.0)	(10.1)	(0.4)
	*cont*	0.3	5.9	30.8	0.0	-1.7	3.3	-8.9	1.2	2.4
	(167)	(2.5)	(5.0)	(3.3)	(0.5)	(0.4)	(0.1)	(5.6)	(10.3)	(0.3)
GG/CC	*free-RNA*	0.5	8.0	31.2	0.1	-1.8	3.3	-10.5	-3.3	2.4
	(157)	(2.6)	(3.7)	(2.7)	(0.5)	(0.4)	(0.1)	(4.9)	(8.3)	(0.3)
	*non-cont*	0.7	7.7	31.0	0.2	-1.9	3.3	-8.7	-4.5	2.5
	(317)	(2.8)	(4.2)	(3.0)	(0.5)	(0.4)	(0.1)	(4.7)	(9.5)	(0.3)
	*cont*	1.1	7.3	31.0	0.2	-1.9	3.3	-8.5	-5.0	2.5
	(488)	(2.7)	(4.1)	(3.2)	(0.5)	(0.4)	(0.1)	(5.6)	(9.8)	(0.3)
RR	*free-RNA*	0.5	8.0	31.6	0.1	-1.5	3.3	-11.6	-1.8	2.2
	(347)	(2.2)	(3.7)	(2.5)	(0.4)	(0.4)	(0.1)	(4.8)	(7.7)	(0.3)
	*non-cont*	0.7	7.4	31.1	0.1	-1.8	3.3	-9.2	-2.7	2.4
	(570)	(2.8)	(4.5)	(3.1)	(0.5)	(0.4)	(0.1)	(5.3)	(9.9)	(0.3)
	*cont*	1.0	7.1	30.9	0.2	-1.8	3.3	-8.8	-2.7	2.4
	(879)	(2.6)	(4.4)	(3.3)	(0.5)	(0.4)	(0.1)	(5.6)	(10.4)	(0.3)
*ModelRNA*		-0.4	13.1	30.0	0.0	-1.2	3.3	-10.4	0.0	2.2

Mean values and SD of dinucleotide step parameters for RR steps along with average propeller-twist (Prop.av), Cup and Zp in *free-RNA*, protein non-contacting (*non-cont*) and contacting (*cont*) datasets. Frequency of occurrence of each dinucleotide step and standard deviation of the parameter values are given in parenthesis. Values of Tilt, Roll, Twist, Cup and Prop.av are in degrees (°), while Shift, Slide, Rise and Zp are in Angstroms (Å).

**Table 5:** Dinucleotide step parameters values for RY steps in RNA helices

**Step**	**Dataset ** **(n)**	**Tilt**	**Roll**	**Twist**	**Shift**	**Slide**	**Rise**	**Prop.av**	**Cup**	**Zp**
AC/GU	*free-RNA*	0.6	6.0	31.8	0.2	-1.3	3.2	-13.4	6.0	2.2
	(96)	(1.9)	(3.4)	(2.8)	(0.5)	(0.3)	(0.1)	(4.9)	(6.3)	(0.2)
	*non-cont*	0.9	5.3	31.5	0.2	-1.3	3.2	-12.2	7.3	2.2
	(76)	(2.4)	(4.9)	(3.0)	(0.5)	(0.5)	(0.1)	(6.0)	(8.1)	(0.4)
	*cont*	0.6	4.5	31.9	0.2	-1.4	3.2	-10.3	7.6	2.3
	(143)	(2.4)	(4.6)	(3.1)	(0.5)	(0.5)	(0.1)	(5.7)	(9.7)	(0.4)
AU/AU	*free-RNA*	0.3	10.2	31.0	0.1	-1.1	3.2	-17.4	4.0	1.9
	(20)	(0.9)	(4.6)	(2.1)	(0.2)	(0.3)	(0.1)	(3.9)	(7.0)	(0.2)
	*non-cont*	-0.3	9.7	32.5	-0.0	-1.2	3.2	-13.8	8.8	2.0
	(18)	(2.8)	(7.8)	(2.9)	(0.4)	(0.3)	(0.1)	(4.3)	(11.1)	(0.3)
	*cont*	-0.2	6.6	31.9	0.0	-1.3	3.2	-12.4	5.1	2.2
	(38)	(1.8)	(5.0)	(2.7)	(0.4)	(0.5)	(0.1)	(5.3)	(6.2)	(0.3)
GC/GC	*free-RNA*	0.1	3.9	31.2	0.0	-1.5	3.2	-10.8	7.3	2.4
	(94)	(1.8)	(3.3)	(2.9)	(0.5)	(0.4)	(0.1)	(4.5)	(5.5)	(0.2)
	*non-cont*	0.0	3.6	31.7	-0.2	-1.5	3.2	-9.0	6.9	2.4
	(100)	(2.2)	(5.0)	(3.0)	(0.5)	(0.5)	(0.1)	(5.7)	(8.5)	(0.4)
	*cont*	-0.2	3.2	31.6	-0.2	-1.6	3.2	-8.2	5.6	2.5
	(174)	(2.4)	(4.2)	(3.2)	(0.5)	(0.5)	(0.1)	(6.2)	(8.9)	(0.3)
RY	*free-RNA*	0.3	5.5	31.4	0.1	-1.4	3.2	-12.6	6.4	2.2
	(210)	(1.8)	(3.9)	(2.8)	(0.5)	(0.4)	(0.1)	(5.0)	(6.1)	(0.3)
	*non-cont*	0.3	4.8	31.7	0.1	-1.4	3.2	-10.7	7.2	2.3
	(194)	(2.4)	(5.5)	(3.0)	(0.5)	(0.5)	(0.1)	(6.0)	(8.6)	(0.4)
	*cont*	0.1	4.1	31.8	0.0	-1.5	3.2	-9.5	6.3	2.4
	(355)	(2.4)	(4.6)	(3.1)	(0.5)	(0.5)	(0.1)	(6.1)	(9.0)	(0.4)
*ModelRNA*		-0.4	13.1	30.0	0.0	-1.2	3.3	-10.4	0.0	2.2

Mean values and SD of dinucleotide step parameters for RY steps along with average propeller-twist (Prop.av), Cup and Zp in *free-RNA*, *non-cont* and *cont* datasets. Other details are same as in Table 4.

**Table 6:** Dinucleotide step parameters values for YR steps in RNA helices

**Step**	**Dataset ** **(n)**	**Tilt**	**Roll**	**Twist**	**Shift**	**Slide**	**Rise**	**Prop.av**	**Cup**	**Zp**
CA/UG	*free-RNA*	-0.6	11.0	31.1	-0.1	-1.6	3.3	-11.5	-5.3	2.3
	(127)	(1.5)	(3.7)	(2.2)	(0.4)	(0.2)	(0.1)	(3.6)	(7.0)	(0.2)
	*non-cont*	-0.1	11.4	29.9	0.0	-1.7	3.3	-9.2	-4.5	2.2
	(88)	(2.5)	(4.7)	(2.7)	(0.5)	(0.3)	(0.2)	(5.5)	(11.5)	(0.3)
	*cont*	0.1	10.7	30.3	-0.0	-1.7	3.3	-9.6	-4.3	2.2
	(148)	(2.3)	(4.8)	(2.7)	(0.5)	(0.3)	(0.1)	(4.6)	(10.0)	(0.3)
CG/CG	*free-RNA*	-0.2	11.5	31.0	-0.2	-1.8	3.3	-11.2	-10.7	2.3
	(84)	(2.0)	(4.3)	(2.7)	(0.5)	(0.3)	(0.1)	(4.0)	(9.2)	(0.3)
	*non-cont*	0.6	12.8	30.1	0.1	-1.9	3.3	-10.9	-13.5	2.4
	(89)	(2.4)	(4.4)	(2.6)	(0.5)	(0.3)	(0.1)	(5.0)	(10.7)	(0.3)
	*cont*	0.1	11.0	29.7	0.0	-1.9	3.3	-9.4	-9.1	2.4
	(157)	(2.4)	(4.4)	(3.1)	(0.5)	(0.3)	(0.1)	(4.9)	(9.9)	(0.3)
UA/UA	*free-RNA*	0.5	14.1	30.7	0.2	-1.5	3.3	-14.0	-3.9	2.0
	(29)	(2.2)	(3.4)	(2.5)	(0.4)	(0.2)	(0.1)	(3.4)	(7.4)	(0.2)
	*non-cont*	-0.7	12.0	30.1	-0.1	-1.6	3.3	-11.0	-6.5	2.1
	(24)	(2.2)	(5.8)	(2.2)	(0.4)	(0.3)	(0.1)	(5.5)	(9.4)	(0.3)
	*cont*	-0.8	11.2	30.4	-0.0	-1.5	3.3	-10.5	-0.3	2.1
	(27)	(3.1)	(3.9)	(2.2)	(0.4)	(0.2)	(0.2)	(4.0)	(11.5)	(0.2)
YR	*free-RNA*	-0.3	11.5	31.0	-0.1	-1.7	3.3	-11.7	-7.0	2.2
	(240)	(1.8)	(4.0)	(2.4)	(0.4)	(0.3)	(0.1)	(3.8)	(8.3)	(0.3)
	*non-cont*	0.1	12.1	30.0	0.0	-1.8	3.3	-10.2	-8.7	2.3
	(201)	(2.5)	(4.8)	(2.6)	(0.5)	(0.3)	(0.1)	(5.3)	(11.7)	(0.3)
	*cont*	-0.0	10.9	30.0	0.0	-1.8	3.3	-9.6	-6.3	2.3
	(332)	(2.4)	(4.5)	(2.9)	(0.5)	(0.3)	(0.1)	(4.7)	(10.5)	(0.3)
*ModelRNA*		-0.4	13.1	30.0	0.0	-1.2	3.3	-10.4	0.0	2.2

Mean values and SD of dinucleotide step parameters for steps along with average propeller-twist (Prop.av), Cup and Zp in *free-RNA*, *non-cont* and *cont* datasets. Other details are same as in Table 4.

In general, average propeller-twist value is higher for dinucleotide steps containing only AU basepairs (AA/UU, AU/AU and UA/UA) and lower for steps with only GC basepairs (GG/CC, GC/GC and CG/ CG). Roll value differs between RR, RY and YR steps (YR > RR > RY), though the overall average roll value for WC basepair containing steps is lower than *ModelRNA* value (Table 4). It is interesting to note that among the RR sequences, the GG/CC step show slightly larger negative slide and positive Zp value, while AA/UU step has the smallest negative slide value, which is also reflected in their smaller Zp value. All other parameters have similar values.

Among RY steps, AC/GU and GC/GC steps have smallest positive roll angles and negative slide values, among all dinucleotide steps (Table 5). However, AU/AU steps have high roll and large negative average propeller-twist and low slide values, with correspondingly small Zp value, as compared to AC/GU and GC/GC steps. This finding was specific to RNA since the equivalent AT/AT steps in both *ADNA* and *BDNA* did not show any such difference when compared to other RY steps. All three YR steps have larger roll and negative slide values when compared to the RR and RY steps, as well as the *ModelRNA* (Table 6). The UA/UA steps show particularly high mean roll angle (14.1°) compared to CA/UG (11.0°) and CG/CG (11.5°). In addition, the slide and cup values for CG/CG steps have larger negative values, than those for CA/UG and UA/UA steps. Most of these sequence dependent features are unique to RNA helices, however the trends observed for them, seem to be similar to those reported earlier for DNA, particularly the trend observed for roll angle values (YR > RR > RY). Hence, an analysis of these trends and correlation between various parameters in *freeRNA*, *ADNA* and *BDNA* datasets has been carried out to identify any specific structure based features.

### Correlation between dinucleotide step parameters

**Figure 1:**
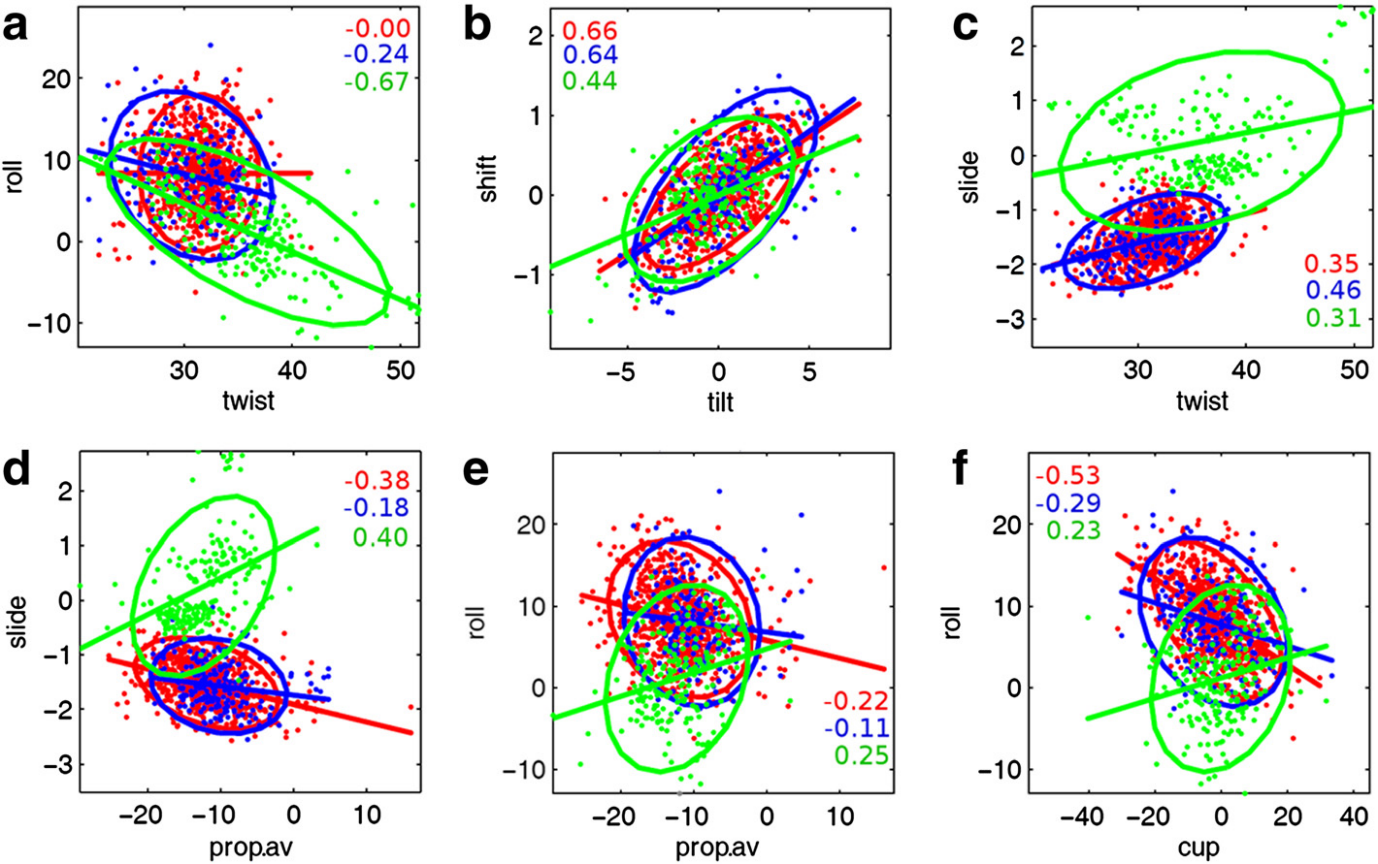
**Correlation between dinucleotide step parameters for Watson-Crick basepair containing steps in *RNA *and *DNA *helices.** The dinucleotide step parameters in each dataset are plotted along with a Mahalanobis ellipse that is fitted with the mean as centre. Correlation coefficient (r) value and best-fit line for each group are also shown. The data are colour coded as red: f*ree-RNA*, blue: *ADNA*, green: *BDNA*. For the sake of clarity, *bound-RNA* dataset is not included here, but shows similar trends as *free-RNA*. Prop.av: corresponds to average propeller-twist of both basepairs constituting a step. Correlations between a few selected parameters are shown here **(a-f)**. See Additional file 1 for the complete data on correlation between all parameters.

We have carried out a pair-wise correlation analysis between the various step parameters in the *free-RNA* dataset and compared the correlation coefficient values (r) with those of *ADNA* and *BDNA* datasets in order to identify correlations that are specific to A-form helices. The parameters that show statistically significant correlations at confidence level > 99.9% being discussed further (Figure 1, Additional file 1). The well-characterized strong correlation between roll and twist that is observed for *BDNA*[3] is not seen for either *ADNA* or *free-RNA* dataset. On the other hand, correlations between shift and tilt and slide and twist are present in both RNA and DNA datasets. Interestingly, the major differences between A and B-form structures are seen for correlations between basepair geometry dependent step parameters and other dinucleotide step parameters. For instance, average-propeller twist is positively correlated with roll, slide and rise in *BDNA*. However, in *ADNA* and RNA datasets it is negatively correlated with roll, twist and slide. Twist shows a significant negative correlation with cup value in *BDNA*, which is absent in A-form structures. Instead, roll and rise show negative correlations with cup in A-form structures, while slide shows a positive correlation with cup. Thus, overall, dinucleotide step parameters in RNA and *ADNA* datasets show similar correlations that are distinct from those in *BDNA* dataset.

**Figure 2:**
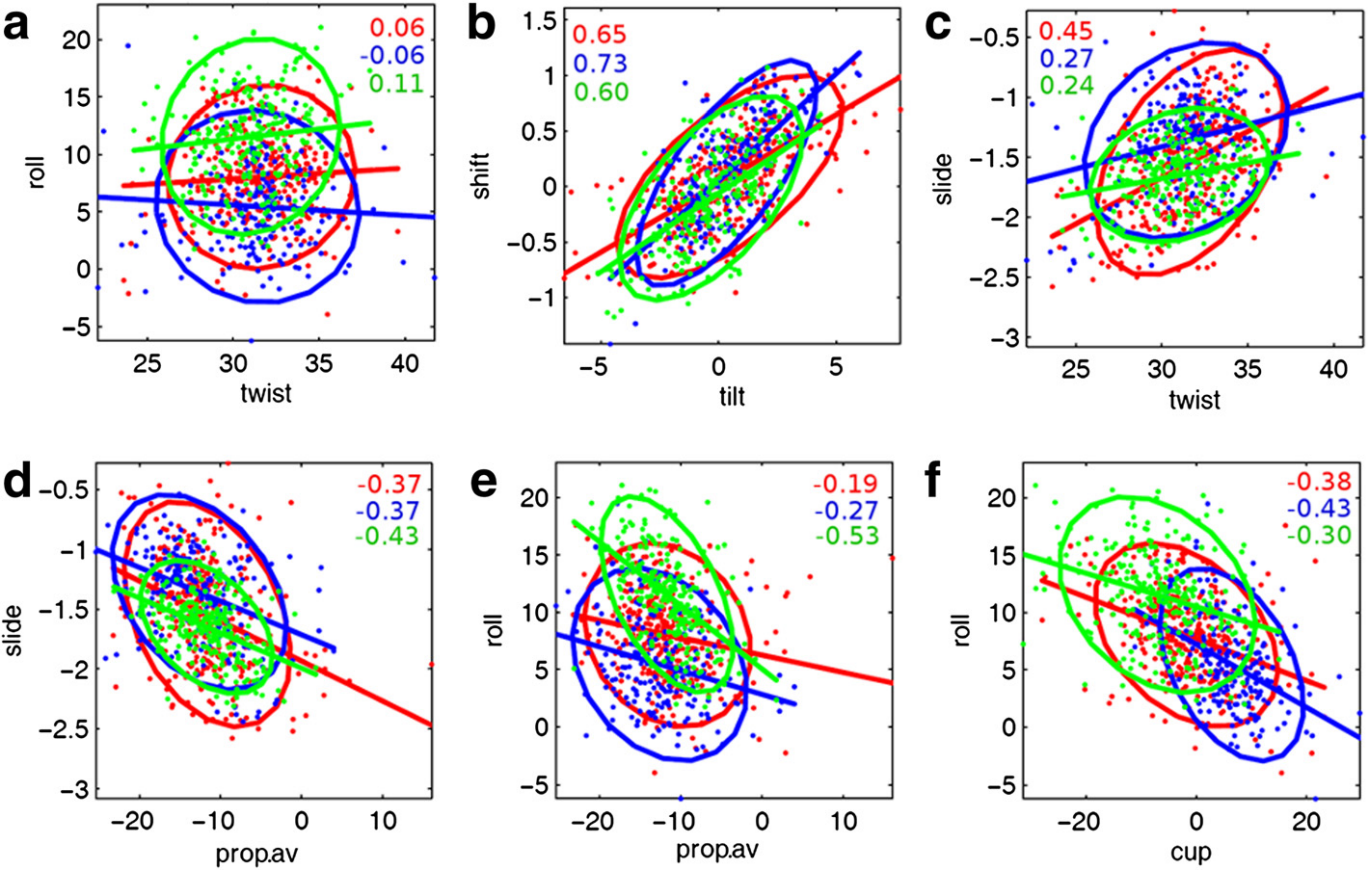
**Correlation between some dinucleotide step parameters for RR, RY and YR type steps in *free-RNA *dataset.** Panels **(a-f)** show the correlations between the same pairs of dinucleotide step parameters as shown in Figure 1. A Mahalanobis ellipse fitted with the mean as centre. Correlation coefficient (r) and best-fit line calculated for each group, are also shown. An 'r' value ≥ 0.14, ≥ 0.18, ≥0.18 is significant at 99.9% confidence level for RR, RY and YR steps respectively. The data points as well as ‘r’ values for RR, RY and YR steps are shown in red: RR (n = 347), blue: RY (n = 210), green: YR (n = 240).

We have also carried out a correlation analysis, for *free-RNA* dataset, considering the dinucleotide steps in each of the three sub-groups, RR, RY and YR separately (Figure 2). No significant correlation is seen between roll and twist, for any of the three sub-groups, confirming that this is absent in RNA. Several correlations between step parameter values showed same trend for RR, RY and YR type of steps, e.g. shift with tilt and slide with twist. Similarly, average propeller-twist shows significant negative correlation with slide and roll for all three dinucleotide step types. Cup also shows a negative correlation with roll in all three sub-groups. Thus, a comparison of correlations between parameters of the three step types suggests that the correlations seen in majority of the step are similar to those seen in the pooled dataset and are characteristic of A-form structure.

### Effect of Protein binding on the dinucleotide step geometry

The mean and standard deviation values of intra-basepair parameters for canonical AU and GC basepairs in the *bound-RNA* dataset were compared with that of *free-RNA* dataset (Table 3). Almost all the parameters have larger standard deviation values in the bound dataset, indicating larger conformational sampling. However, mean values of intra-basepair parameters in b*ound-RNA* dataset show no significant difference from *free-RNA* values, except for propeller-twist values. Though well within standard deviation, basepair propeller-twist in *bound-RNA* (AU: - 9.5°; GC: - 8.9°) have smaller negative values compared to *free-RNA* (AU: -12.7°; GC: - 10.9°).

Approximately 62% of the total dinucleotide steps in *bound-RNA* dataset are in direct contact with protein (Table 2). To examine the direct and indirect effect of protein binding, the dataset was divided into two sub-datasets: those steps that are in contact with protein (*cont*) and those that do not contact the protein (*non-cont*). The mean and standard deviation values of the step parameters and the corresponding average propeller-twist, cup and Zp value, for each of the 10 dinucleotide steps in the *non-cont* and *cont* dataset are tabulated separately in Tables 4, 5 and 6. The mean values of steps parameters of *non-cont* and *cont* dataset are quite similar to each other, but differ slightly from *free-RNA* values. Interestingly both *cont* and *non-cont* data show large standard deviation values. In addition, the correlation analysis for dinucleotide steps in the *bound-RNA* dataset does not show any significant difference from that of *free-RNA* and *ADNA* datasets (Additional file 1).

**Figure 3:**
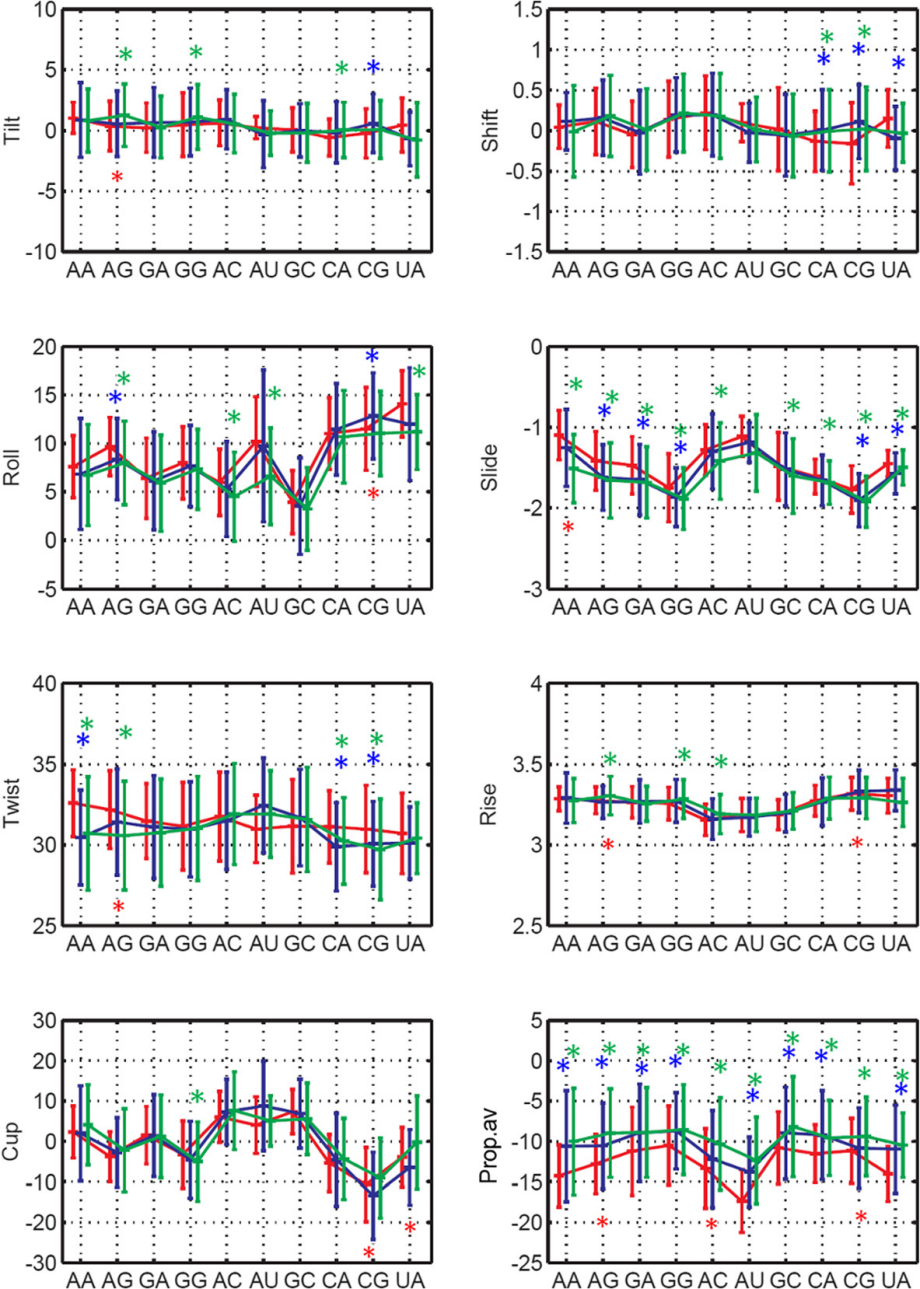
**Comparison of WC dinucleotide step parameters between *free-RNA*, *cont *and *non-cont *RNA datasets.** The mean values and standard deviation (±1σ) of all the parameters are plotted. The mean of step parameters are connected by a line in all three datasets and are colour coded as Red: free-RNA; Blue: non-cont; Green: cont. Parameters that differ significantly between two datasets (with P < 0.05) are marked by ‘*’ in Red for non-cont and cont, in Blue for *free-RNA* and *non-cont* and in Green for *free-RNA* and *cont datasets.*

To check for statistical significance of the differences between the step parameters of the three datasets (*free-RNA*, *non-cont* and *cont*) unpaired student t-test was carried out (Figure 3). No significant difference was found between *cont* and *non-cont* datasets. However, roll, slide and average propeller-twist values for several of the dinucleotide steps in the *cont* dataset show significant difference (P value < 0.05) from the *free-RNA* dataset. Some of the steps also showed significant difference between *free-RNA* and *non-cont* dataset.

### Base overlap and formation of non-canonical hydrogen bonds in dinucleotide steps

**Figure 4:**
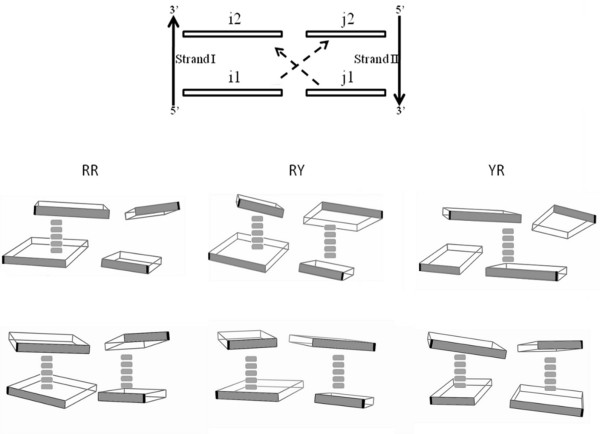
**Schematic diagrams showing the nomenclature used and block diagrams illustrating the major base overlaps in A-RNA and BDNA.** The mutual overlap between bases i1-i2 and j1-j2 represent intra-strand overlap, while that between i1-j2 and j1-i2 correspond to cross-strand overlap. The blocks are drawn with the minor groove facing edge of each base shaded grey and the large blocks representing purines. The glycosidic bond attachment point is marked in black. The distinct stacking pattern of bases in RR, RY and YR steps is shown for RNA (Row 1) and BDNA (Row 2). A thick dashed line is drawn connecting the bases that show significant overlap. The base coordinates are taken from representative crystal structures (PDB_ID: 1RNA and 1BNA) and block diagrams drawn using 3DNA program [42].

Since the parameters for RR, RY and YR steps show some significant differences from *Model-RNA*, we calculated base stacking overlap for these steps and compared it for the various crystal datasets and the corresponding model structures (Table 7). Figure 4 illustrates the nomenclature used to refer the bases involved in basepair overlap area calculation and the different stacking patterns for RR, RY and YR steps, in A and B-form structures. In case of *BDNA* dataset*,* all three types of steps show only intra-strand base overlap, but negligible cross-strand overlap, with RR > RY > YR and the major contribution coming from exo-cyclic atoms, in all cases. In general, the overlap increases in the crystal structure steps as compared to the *fibre-BDNA* model. In A-form helices, the RR, RY and YR steps show distinctly different features, with RY> > RR ≈ YR. In RR steps, high intra-strand base overlap is seen in strand I (Pur-Pur stacking) while, unlike in *BDNA*, there is very little overlap in strand II (Pyr-Pyr stacking) and no overlap between the cross-strand bases. RY steps show high intra-strand base overlap along both strands I and II and no cross-strand overlap, with the exo-cyclic atoms making substantial contribution. YR steps in RNA and *ADNA* datasets are characterized by very small intra-strand contribution and stacking arises mainly due to cross-strand overlap of purine bases, with contributions from both ring and exo-cyclic atoms. Interestingly the overlap in *free-RNA* crystal structure steps is smaller than in the *ModelRNA*, for RR and RY steps. Our findings suggest that the combined effect of large negative slide and lower twist value contribute towards these overlap patterns, indicating that the interactions that determine the base stacking preferences of dinucleotide steps in RNA helices are different from those seen in DNA helices. We have therefore analyzed various dinucleotide steps to see whether the base overlap patterns are related to formation of some potential non-canonical, intra-strand or cross-strand hydrogen bonds.

**Table 7:** Average stacking area overlap for dinucleotide steps in crystal structure datasets and fibre models

**Dataset/Model**	**Intra-strand**		**Cross-strand**		**Cross-strand**		**Intra-strand**		**Total**	
	**(i1-i2)**		**(i1-j2)**		**(j1-i2)**		**(j1-j2)**			
	**All**	**Ring**	**All**	**Ring**	**All**	**Ring**	**All**	**Ring**	**All-atom**	**Ring**
**RR**										
*ModelRNA*	3.1 (0.7)	2.4 (0.1)	0.0 (0.0)	0.0 (0.0)	0.0 (0.0)	0.0 (0.0)	1.0 (0.0)	0.1 (0.0)	4.1 (0.7)	2.4 (0.1)
*freeRNA (347)*	3.0 (1.1)	1.9 (0.8)	0.1 (0.3)	0.0 (0.0)	0.2 (0.3)	0.0 (0.0)	0.6 (0.7)	0.1 (0.2)	3.8 (1.1)	2.0 (0.8)
*ADNA (94)*	3.4 (1.1)	2.0 (1.0)	0.1 (0.2)	0.0 (0.0)	0.1 (0.2)	0.0 (0.0)	0.6 (1.0)	0.1 (0.3)	4.3 (1.0)	2.1 (0.8)
*fibre-BDNA*	4.4 (0.9)	2.1 (0.1)	0.0 (0.0)	0.0 (0.0)	0.0 (0.0)	0.0 (0.0)	4.8 (1.1)	0.2 (0.0)	9.2 (0.2)	2.3 (0.1)
*BDNA (107)*	4.2 (1.6)	2.1 (1.3)	0.0 (0.0)	0.0 (0.0)	0.0 (0.0)	0.0 (0.0)	4.6 (1.4)	0.4 (0.5)	8.8 (1.8)	2.5 (1.2)
**RY**										
*ModelRNA*	5.2 (0.9)	3.1 (0.1)	0.0 (0.0)	0.0 (0.0)	0.0 (0.0)	0.0 (0.0)	5.7 (0.9)	3.2 (0.1)	10.9 (1.5)	6.4 (0.1)
*freeRNA (210)*	5.1 (1.1)	2.9 (0.9)	0.0 (0.0)	0.0 (0.0)	0.0 (0.0)	0.0 (0.0)	5.4 (1.3)	2.7 (1.1)	10.5 (1.5)	5.6 (1.0)
*ADNA (42)*	5.4 (1.1)	2.9 (1.0)	0.0 (0.0)	0.0 (0.0)	0.0 (0.0)	0.0 (0.0)	6.2 (1.2)	2.7 (1.0)	11.6 (0.9)	5.6 (0.7)
*fibre-BDNA*	3.0 (0.8)	0.4 (0.0)	0.0 (0.0)	0.0 (0.0)	0.0 (0.0)	0.0 (0.0)	3.6 (1.2)	0.4 (0.0)	6.6 (1.4)	0.9 (0.0)
*BDNA (48)*	4.2 (2.0)	1.6 (1.1)	0.0 (0.2)	0.0 (0.0)	0.0 (0.0)	0.0 (0.0)	4.9 (1.6)	1.6 (0.9)	9.1 (2.8)	3.2 (0.7)
**YR**										
*ModelRNA*	0.4 (0.0)	0.0 (0.0)	0.0 (0.0)	0.0 (0.0)	2.3 (1.1)	0.9 (0.0)	0.4 (0.0)	0.0 (0.0)	3.0 (1.1)	0.9 (0.0)
*freeRNA (240)*	0.1 (0.2)	0.0 (0.0)	0.8 (1.3)	0.4 (0.6)	2.3 (1.8)	1.0 (0.7)	0.2 (0.3)	0.0 (0.1)	3.4 (0.9)	1.4 (0.4)
*ADNA (59)*	0.3 (0.5)	0.1 (0.2)	0.2 (0.7)	0.1 (0.3)	3.2 (1.7)	1.2 (0.8)	0.3 (0.4)	0.0 (0.1)	4.0 (0.9)	1.4 (0.6)
*fibre-BDNA*	1.5 (0.0)	0.0 (0.0)	0.0 (0.0)	0.0 (0.0)	0.2 (0.3)	0.0 (0.0)	1.5 (0.0)	0.0 (0.0)	3.2 (0.3)	0.0 (0.0)
*BDNA (57)*	2.2 (1.5)	0.1 (0.3)	0.1 (0.2)	0.0 (0.0)	0.2 (0.5)	0.0 (0.0)	2.6 (1.8)	0.2 (0.4)	5.0 (2.0)	0.4 (0.5)

The stacking area overlap was grouped for RR, RY and YR steps in free RNA, ADNA and BDNA datasets and for A and B-form fibre models. Two sets of calculations were carried out by considering: i) all atoms including ring and exocyclic groups in each nucleotide base and ii) only ring atoms. Both intra-strand and cross-strand overlap areas are listed. Units are in Å^2^. See Figure 4 for the nomenclature used to refer to intra and inter-strand base overlaps.

**Figure 5:**
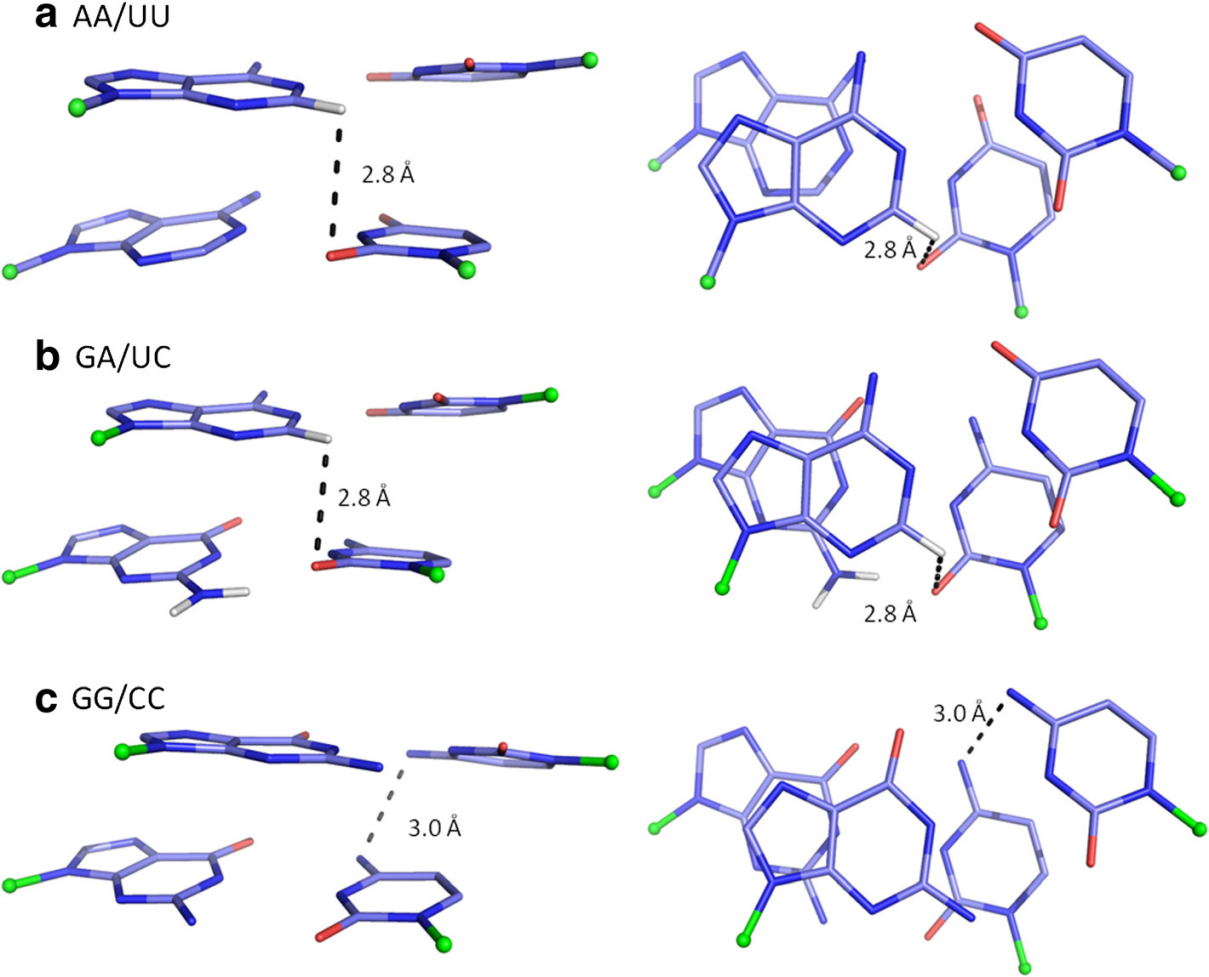
**Stick drawings of representative dinucleotide steps favouring cross-strand and intra-strand hydrogen bonds in RR steps.** C1’ atoms are represented as green balls. Edge on view from minor groove side and projection down the z-axis are shown in each case. **a)** A representative AA/UU and **b)** GA/UC step with C-H..O cross-strand hydrogen bond. The distances between 3′-Ade-H2 and 3′-Ura-O2/3′-Cyt-O2 are marked. **c)** A GG/CC step with intra-strand N-H..N interaction between 5′-Cyt-N4 and 3′-Cyt-N4 atoms is shown with the N4..N4 distance being marked. See Additional file 2 for details of the structures selected and hydrogen bond parameters.

**Figure 6:**
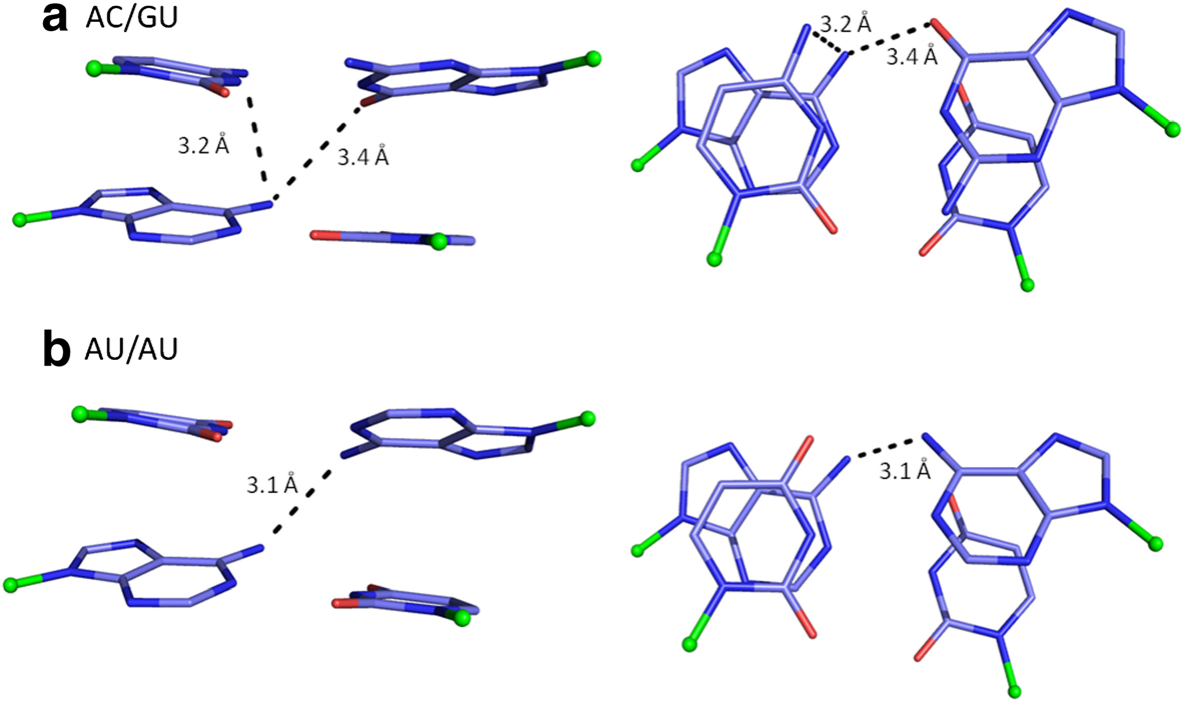
**Stick drawings of dinucleotide steps favouring cross-strand and intra-strand hydrogen bonds in RY steps. a)** AC/GU step with N-H..N intra-strand and N-H..O cross-strand hydrogen bonds is shown. The distance between 3′-Cyt-N4 and 5′-Ade-N6, as well as 5′-Ade-N6 and 5′-Gua-O6 are marked. **b)** AU/AU step, showing an N-H..N cross-strand hydrogen bond between the N6 groups of both 5′-Ade. Other details are as in Figure 5. See Additional file 2 for details of the structures selected and hydrogen bond parameters.

**Figure 7:**
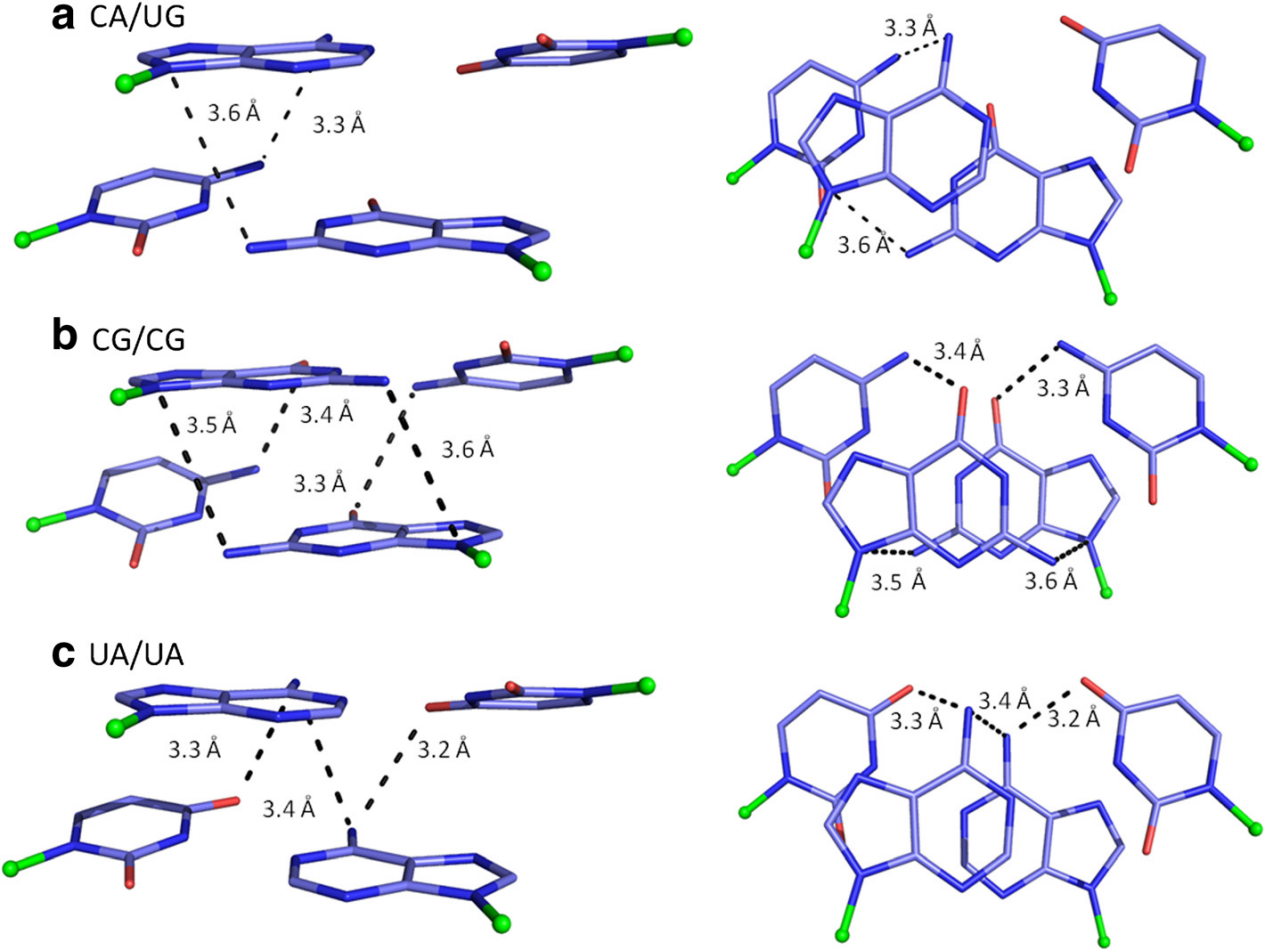
**Stick drawings of dinucleotide steps favouring cross-strand and intra-strand hydrogen bonds in YR steps. a)** In CA/UG step, an N-H..N intra-strand hydrogen bond is shown, with the distance between 3′-Ade-N6 and 5′-Cyt-N4 indicated. Also, an unusual N-H..N cross-strand hydrogen bond is observed between 3′-Gua-N2 and 3′-Ade-N9. **b)** In CG/CG step, two N-H..O intra-strand hydrogen bonds are shown. The distance between 5′-Cyt-N4 and 3′-Gua-O6 in each strand is marked. Also, two N-H..N cross-strand hydrogen bonds are shown. The distance between strand II, 3′-Gua-N2 (donor) and strand I,3′-Gua-N9 (acceptor) is marked. Similarly, distance between strand I, 3′-Gua-N2 (donor) and strand II, 3′-Gua-N9 (acceptor) is marked. **c)** In UA/UA step, two N-H..O intra-strand hydrogen bonds are observed. The distance between 3′-Ade-N6 and 5′-Ura-O4 is marked in each strand. In addition, an N-H..N cross-strand hydrogen bond is shown between the two 3′-Ade-N6 groups. Other details are as in Figure 5. See Additional file 2 for details on the structures selected and hydrogen bond parameters.

Some of the geometric preferences seen in dinucleotide steps of DNA have been attributed to the presence of additional hydrogen bond interactions between the bases, particularly in oligo-A tracts [30,34,35]. Similarly, non-canonical hydrogen bonds between RNA bases involved in forming a dinucleotide steps can arise due to favourable intra-strand or cross-strand interactions on both major groove and minor groove side. Many such potential hydrogen bonds are possible in RNA model structure and are found to occur in crystal structures, but only those interactions that occur in more than 50% of each of the steps are discussed here. A list of such cross-strand and intra-strand interactions, along with the mean values of donor-acceptor (DA) distance, hydrogen-acceptor distance (HA) and hydrogen bond angle (DHA) in each dinucleotide step in *free-RNA* dataset is given in Table 8. Stick drawings of dinucleotide steps with hydrogen bonds marked for selected example (Additional file 2) from crystal structures for RR, RY and YR steps are shown in Figures 5, 6, and 7 respectively. A complete list of hydrogen bonds present identified in RNA and DNA crystal datasets and fibre model structures is given in Additional file 3. It is observed that the number of non-canonical hydrogen bonds is more in *ModelRNA* as compared to *fibre-BDNA* model. Many of these are retained, with improved hydrogen bond parameters, in the RNA crystal structures, while some potential interactions are found to occur in specific dinucleotide steps.

**Table 8:** **Non Watson-Crick hydrogen bonds commonly observed in *free-RNA *** **helices**

**Step**	**Cross/Intra-strand**	**Groove face**	**Base (atom) involved**	**DA**	**HA**	**DHA**
			**Donor, Acceptor**	**(Å)**	**(Å)**	**( ° )**
**RR**						
AA/UU	Cross	m	A(C2,H2),U(O2)	3.4(0.2)	3.0(0.2)	101.3(7.5)
GA/UC	Cross	M	A(C2,H2),U(O2)	3.4(0.3)	3.0(0.3)	97.8(6.4)
GG/CC	Intra	M	C(N4,H41),C(N4)	3.4(0.2)	3.0(0.2)	101.3(7.9)
**RY**						
AC/GU	Intra	M	C(N4,H42),A(N6)	3.3(0.1)	3.1(0.2)	95.1(5.1)
AC/GU	Cross	M	A(N6,H61),G(O6)	3.4(0.2)	2.9(0.2)	114.8(6.1)
AU/AU	Cross	M	A(N6,H61),A(N6)	3.2(0.2)	2.7(0.2)	114.8(5.5)
**YR**						
CA/UG	Intra	M	A(N6,H62),C(N4)	3.4(0.2)	2.9(0.2)	108.7(6.5)
CA/UG	Cross	m	G(N2,H22),A(N9)	3.6(0.1)	3.3(0.1)	98.2(4.6)
**C**G/CG*	Intra	M	C(N4,H42),G(O6)	3.5(0.2)	3.1(0.2)	106.8((7.6)
CG/**C**G*	Intra	M	C(N4,H42),G(O6)	3.5(0.2)	3.1(0.2)	104.1(6.7)
C**G**/CG*	Cross	m	G(N2,H22),G(N9)	3.6(0.1)	3.3(0.1)	98.0(5.0)
CG/CG*	Cross	m	G(N2,H22),G(N9)	3.6(0.1)	3.3(0.1)	96.2(4.6)
U**A**/UA*	Intra	M	A(N6,H62),U(O4)	3.3(0.2)	2.9(0.2)	104.6(6.2)
UA/UA*	Intra	M	A(N6,H62),U(O4)	3.3(0.2)	2.8(0.2)	108.1(7.7)
UA/UA	Cross	M	A(N6,H61),A(N6)	3.5(0.2)	3.2(0.2)	97.1(3.5)

Mean and SD values of distances between hydrogen bond donor (D)-acceptor (A) atoms and DHA angles, which are observed in more than 50% of each of the 10 dinucleotide steps. Both intra-strand and cross-strand hydrogen bonds on "Minor groove face" (m) and "Major groove face" (M) are listed.

*In steps containing two hydrogen bonds with identical donor-acceptor pairs, the base contributing the donor atom is shown in bold.

Among RR steps, cross-strand C-H..O hydrogen bonds are found in 85% and 59% of AA/UU and GA/UC steps respectively in *freeRNA*, on the minor groove side (Figure 5 and Additional file 3). Similar interaction is also observed, though in smaller numbers, for bound RNA steps (*non-cont* and *cont*), as well as AA/TT and GA/TC steps in *BDNA.* The cross-strand N-H..O interaction between 6-amino group of Adenine and O4 atom of Uracil that is commonly seen in AA/TT steps of *BDNA* (89%) is favoured only in 40% of the AA/UU steps in *freeRNA* dataset. In GG/CC steps, intra-strand N-H..N interaction between the two Cytosine 4-amino groups shows significant presence in all A-form structures. It is not present in *fibre-BDNA* model but is seen in *BDNA* crystal structures with a slightly longer donor-acceptor (DA) distance, as compared to RNA structures. Though a similar pair of 6-amino groups of Adenine is present in AA/UU, they do not have favourable hydrogen bond geometry.

RY steps in A-form helices are characterized by large intra-strand overlap and the exo-cyclic atoms in the major groove are positioned almost above each other, leading to unfavorable N-H..O angles, in both AU/AU and GC/GC steps. However, AC/GU step show presence of a weak intra-strand N-H..N interaction between the exo-cyclic amino groups in Adenine (N6) and Cytosine (N4) (Figure 6 and Additional file 3). This interaction is seen in RNA as well as in DNA helices, though the *ModelRNA* and *fibre-BDNA* model does not have a favourable geometry for this hydrogen bond. In addition, cross-strand interactions between the two purine bases are highly favoured in AC/GU and AU/AU steps in all RNA datasets and equivalent steps in DNA. A cross-strand N-H..O hydrogen bond is present in 83% of AC/GU in *free-RNA*, between 6-amino group of Adenine and O6 atom of Guanine. An even larger number (~90%) of AU/AU steps show cross-strand N-H..N interaction between the 6-amino group of Adenines, in both *free-RNA* and *BDNA* datasets. A combination of high negative average propeller-twist, smaller slide and positive roll values, seen in AU/AU steps in RNA favors this cross-strand interaction, while the intra-strand N-H..O hydrogen bond is relatively infrequent. GC/GC step does not show significant occurrence of any intra-strand or cross-strand interaction between their exo-cyclic groups.

In A-form helices, the large negative slide leads to high cross-strand overlap between the purine bases in YR steps (Table 7). Interestingly, the relative displacement of neighbouring bases within a strand, along with large positive roll, gives rise to favourable orientation of exo-cyclic groups in the A-form structure and hence all possible N-H..O and N-H..N hydrogen bonds are seen in large numbers. An intra-strand N-H..N interaction is present in CA/UG step between the 6-amino group of Adenine and 4-amino group of Cytosine; in 91% of the steps in *freeRNA* (Figure 7 and Additional file 3). However, the relative orientation of the 6-amino group of Adenine and O6 oxygen atom of Guanine in the major groove does not favour a cross-strand hydrogen bond between them. Intra-strand N-H..O interaction between the 4-amino group of Cytosine and O6 oxygen atom of Guanine is seen in >60% of CG/CG steps, in both strands of RNA structures, while they are absent in *BDNA* dataset. Almost 100% of UA/UA steps in *freeRNA* and 80-95% in protein bound-RNA helices, form intra-strand N-H..O interaction between 6-amino group of Adenine and O4 oxygen atom of Uracil. A cross-strand N-H..N interaction between the two 6- amino groups of Adenine is also present in more than 60% of the A-like steps.

A rather unusual cross-strand N-H..N interaction is frequently observed between the 2-amino group of Guanine and N9 atom of the Purine base in CA/UG and CG/CG steps in A-like structures (Table 8 and Additional file 3). Unlike other hydrogen bonds that are present in both model structure and RNA datasets, these N2..N9 interactions are much more favourable in the crystal dataset, with the mean Donor-Acceptor distance (DA) being ~3.6 Å (while it is ~4.1 Å in *ModelRNA* structure). This type of hydrogen bond is observed in ~65% of CA/UG steps, (Figure 7a). A similar type of hydrogen bond is seen in ~50% of CG/CG steps, with 31% showing a pair of reciprocal hydrogen bonds (Figure 7b). A combination of relatively higher values for negative cup, negative propeller twist and negative slide is characteristic of steps with reciprocal interactions between the 2-amino groups and N9 atoms of Guanines in CG/CG steps.

Thus, a number of non-canonical hydrogen bonds are present in the WC steps of both free as well as bound RNA crystal structures and their presence can be related to the sequence dependent geometries seen in the various dinucleotide steps. Overall, the percentage occurrence of these non-WC hydrogen bonds is smaller in bound dataset compared to *free-RNA* dataset.

## Discussion

Contrary to the generally accepted view that RNA helices are uniform and rigid, the various dinucleotide steps have characteristic features and can contribute to heterogeneity in the RNA helical regions. The intra-basepair parameters, propeller-twist and buckle, of the AU and GC basepairs show the usual preferences (large propeller-twist in AT and larger buckle in GC basepairs), which can influence the dinucleotide step geometry. Roll values differentiate the three types of steps in RNA. RY steps have small roll (except for AU/AU steps), YR have high roll and RR have intermediate roll values. Interestingly, while roll values in DNA vary from small negative to small positive, a similar trend is seen with roll for YR > RR > RY steps. In B-DNA, the difference in parameters between RR, RY and YR is attributed to the effect of exocyclic groups on slide, roll and twist. Unlike the dinucleotide steps in B-DNA, which show a large variation in twist value that is strongly correlated with roll and moderately with slide, the twist values of steps in RNA helices cluster within a small range and show a significant correlation only with slide. The larger positive roll and negative slide values lead to a large number of favourable intra as well as cross-strand interactions involving the exo-cyclic groups, particularly in YR steps. Interestingly the slide values of WC steps show a significant negative correlation with average propeller-twist in RNA and a positive correlation for B-DNA. Steps with large average propeller-twist (AA/UU, AU/AU and UA/UA) have smaller negative slide values in RNA. The proximity of atoms on major groove side, arising due to high roll and negative propeller-twist prevents large negative slide. Thus, the slide parameter is directly influenced by propeller-twist of the constituent basepairs, but as the basepairs become near planar (average propeller twist ≈ 0°), slide becomes large negative in RNA and small positive in B-DNA steps. Overall, the six dinucleotide step parameters in RNA helices show mean values as well as correlated variations that are different from those observed in B-form DNA [2,4,6,19,49].

The specificity in RNA-protein interaction is thought to be mainly brought about by the exposed bases [15] which are present at helix termini, in bulges and loops. In our analysis, we find that the interactions occur mainly with the phosphate backbones and very few interactions are seen between proteins and the base atoms. In our study, majority of steps in the protein contacting (*cont)* and non-contacting (*non-cont)* dataset, showed statistically significant difference in comparison to *free-RNA* dataset for roll, slide and average propeller-twist. This suggests that, apart from protein induced conformation change on direct contact, it can allosterically affect the steps that are not in direct physical contact.

Recently, various additional hydrogen bond interactions have been reported from the RNA double helical regions, between the base and phosphate group oxygens (BPh) [26,27,43]. Also, the presence of weak hydrogen bonds between cross-strand amino groups in AA/TT and GA/TC steps of B-form DNA are well documented in crystal structure [50] and supported by theoretical quantum chemical calculations [51]. Similarly the presence of C-H..O interactions were reported in B-DNA crystal structures [30]. The presence of cross-strand C-H..O interactions in AA/UU and GA/UC steps and N-H..N interaction in AU/AU steps have been reported from MD simulations of A-RNA duplex sequences [36]. Two other cross-strand N-H..O interactions on the minor groove side in AG/CU and GG/CC steps, reported in the MD studies, occur in less than 20% of these steps in our RNA dataset. However, our analysis has confirmed the presence of other cross-strand interactions and identified novel cross-strand and intra-strand hydrogen bonds that can potentially provide added stability to the RNA dinucleotide step. The cross-strand C-H..O interaction in AT basepair containing steps, AA/TT and GA/TC, reported on the minor groove side of B-DNA crystal structures [29] are surprisingly also found to occur in a majority of AA/UU and GA/UC steps in RNA. In B-DNA helices the AA/TT and GA/TC steps have large negative average propeller twist, but near zero roll and slide, while the AA/UU and GA/UC steps in RNA have large negative average propeller-twist, but moderately positive roll and large negative slide values. Thus, it appears that the same interaction is brought about by a combination of high negative propeller-twist and two different roll-slide geometries, both of which bring the pairing atoms close. Similarly, intra-strand N-H..N interactions in GG/CC and AC/GU steps, cross-strand N-H..O interactions in AC/GU step and the cross-strand N-H..N interactions in AU/AU step are present in both A-form and B-form helices. In RNA, when compared to RR and RY steps, the YR steps show a larger number of these potential hydrogen bonds, due to their unique cross-strand stacking. The weak interaction identified in this study can contribute to stacking and thus to overall stability of the steps. This is in agreement with the results of stacking energy calculated using QM, where the values for RY and YR steps are comparable though the base overlaps as shown in Table 7 are considerably lower for YR steps.

Among YR steps, CG/CG and CA/UG steps, in addition to intra-strand N-H..O and N-H..N hydrogen bonds, they also have an unusual N-H..N interaction between 2-amino groups of Guanines and N9 atoms of Purine bases in these dinucleotide steps. Hydrogen bonds are generally associated with the electronegative character of the donor and acceptor atoms. Electrostatic potential (ESP) derived charges, as well as partial charges in AMBER and CHARMM force fields assign near zero charges to the N9 atoms in Adenosine and Guanosine [52-54]. However partial charges calculated for Adenosine and Guanosine using Natural Bond Orbital (NBO) analysis [55] indicate that the N9 atom is quite negative (Additional file 4). Thus, the presence of this potential hydrogen bond needs to be further examined by quantum chemical methods.

It should also be mentioned that x-ray determined crystal structures do not have coordinates of hydrogen atoms. Programs that add hydrogen atoms to the nucleotide ring atoms as well as the N atoms in the pendent amino group, place the hydrogen atoms in the plane of the base, though many QM studies suggest that these amino groups can have pyramidal geometry with hydrogen atoms being out-of-plane [35,56-59]. The introduction of non-planar pyramidal amino hydrogen atoms can facilitate further improvement in the geometry of N-H..N as well as N-H..O hydrogen bonds discussed here. The hydrogen bonds reported here are more prevalent in free RNA structures than protein-bound RNA helices, where they may be replaced by interactions with proteins or nearby water molecules. Flanking basepairs can also affect the formation of these weak hydrogen bonds. This sequence dependency can be studied by analysing all possible tetramer sequences in helices with the dinucleotide step of interest in the centre. However, the currently available RNA crystal structures do not have sufficient representation of all possible tetramer sequences, for a meaningful analysis.

Apart from using the well-known Watson-Crick edge, a base can pair with other bases using the Hoogsteen or Sugar edges. In our crystal dataset we focused only on the dinucleotide steps formed by WC basepair that belong to the cisWW family (~74%), to compare with equivalent dinucleotide steps in DNA helices. However, more than 32 types of cisWW steps, containing at least one non-Watson-Crick basepair, such as GU, AG and UU, are also present and constitute ~17% of the total number of steps, while ~9% of the steps contain bases that are paired along the Hoogsteen or Sugar edge. Hence, to get a complete picture of the RNA helical geometries, the non-canonical basepair containing steps were analysed, but the small number present for each of these step types in crystal structures poses a challenge in arriving at any statistically significant result.

## Conclusions

Our analysis of the non-redundant RNA crystal structures shows that the RNA dinucleotide steps have characteristic sequence dependent variations. Overall, the steps show features that are attributes of their being of RR, RY or YR type. Several cross-strand and intra-strand potential hydrogen bonds are found to be highly prevalent in RNA helices and can be attributed to the observed geometrical preferences of various dinucleotide steps. Unusual cross-strand interactions are found to be present in the CA/UG and CG/CG steps, between 2-amino groups of Guanines and N9 atoms of Purine bases that are associated with the unique geometry of YR steps. The various dinucleotide steps in RNA bound to proteins show some significant differences in their dinucleotide parameters, from those in free RNA, while retaining most of the gross features, as well as the non-canonical cross-strand and intra-strand interactions.

## Competing interests

The authors declare that they have no competing interest.

## Authors’ contributions

DB and MB conceived the project. SK carried out the analysis. All authors participated in the writing of the manuscript. All authors read and approved the final manuscript.

## Supplementary Material

Additional file 1 **Overall cross-correlation between dinucleotide step parameters for all steps comprising of canonical WC basepairs.** For *free-RNA* dataset (N = 797): correlation coefficient (r) values ≥ 0.15 are significant at 99.9% confidence level. For *bound-RNA* dataset (N = 2531): correlation coefficients (r) values ≥ 0.10 are significant at 99.9% confidence level. For *ADNA* dataset (N = 195): correlation coefficients (r) values ≥ 0.32 are significant at 99.9%. For *BDNA* dataset (N = 212): correlation coefficients (r) values ≥ 0.23 are significant at 99.9% confidence level. ‘r’ values significant at 99.9% level, in each dataset, are shown in bold. Click here for file

Additional file 2 **Details of the structures from which representative RR, RY and YR steps are taken in Figures** 5**,**6**, and**7**.** The PDB IDs, residue ids, bases and atoms involved in intra-strand and cross-strand hydrogen bonding, as well as DA, HA distances and DHA angle are listed for each step. Distances marked in the figures are shown in bold. Click here for file

Additional file 3 **Comparison of non Watson-Crick hydrogen bonds observed in all RNA and DNA helices.** Mean and SD values of donor-acceptor distances and angles observed for cross-strand and intra-strand hydrogen bond in more than 50% of the 10 dinucleotide steps are listed. Hydrogen bond parameters in various model structures are also given. Click here for file

Additional file 4 **Partial charges assigned to atoms in Adenine and Guanine nucleosides.** The partial charges derived from Natural Bond Orbital (NBO) and Electrostatic potential (ESP) calculations are listed, along with those used in AMBER and CHARMM force fields. The large negative charge assigned by NBO calculation is highlighted in bold for the N9 atoms of adenine and guanine bases, which are involved in an unusual cross-strand hydrogen bond with the 2-amino group of guanine in the CA/UG and CG/CG steps respectively (shown in Figure 7a and b). Click here for file
