# Supplementary material for: Sequence dependent variations in RNA duplex are related to non-canonical hydrogen bond interactions in dinucleotide steps
Source: BMC Res Notes. 2014 Feb 7;7:83. doi: 10.1186/1756-0500-7-83 (PMC3930292; doi:10.1186/1756-0500-7-83)
Supplement: Additional file 1 — Overall cross-correlation between dinucleotide step parameters for all steps comprising of canonical WC basepairs. For free-RNA dataset (N = 797): correlation coefficient (r) values ≥ 0.15 are significant at 99.9% confidence level. For bound-RNA dataset (N = 2531): correlation coefficients (r) values ≥ 0.10 are significant at 99.9% confidence level. For ADNA dataset (N = 195): correlation coefficients (r) values ≥ 0.32 are significant at 99.9%. For BDNA dataset (N = 212): correlation coefficients (r) values ≥ 0.23 are significant at 99.9% confidence level. ‘r’ values significant at 99.9% level, in each dataset, are shown in bold. [file 1756-0500-7-83-S1.doc]

**Additional file 1 – Overall cross-correlation between dinucleotide step parameters for all steps comprising of canonical WC basepairs.**

For *free-RNA* dataset (N=797): correlation coefficient (r) values ≥ 0.15 are significant at 99.9 % confidence level. For *bound-RNA* dataset (N=2531): correlation coefficients (r) values ≥ 0.10 are significant at 99.9 % confidence level. For *ADNA* dataset (N=195): correlation coefficients (r) values ≥ 0.32 are significant at 99.9 %. For *BDNA* dataset (N=212): correlation coefficients (r) values ≥ 0.23 are significant at 99.9 % confidence level. ‘r’ values significant at 99.9% level, in each dataset, are shown in bold. (DOCX format)

| ***bound-RNA***  ***free-RNA*** | ***Tilt*** | ***Roll*** | ***Twist*** | ***Shift*** | ***Slide*** | ***Rise*** | ***Prop.av*** | ***Cup*** |
| --- | --- | --- | --- | --- | --- | --- | --- | --- |
| ***Tilt*** |  | 0.04 | 0.01 | **0.57** | -0.10 | **0.11** | 0.00 | **-0.16** |
| ***Roll*** | -0.02 |  | 0.01 | 0.05 | 0.05 | 0.07 | **-0.21** | **-0.52** |
| ***Twist*** | 0.00 | 0.00 |  | -0.08 | **0.35** | **-0.12** | **-0.25** | **-0.16** |
| ***Shift*** | **0.66** | -0.02 | -0.02 |  | **-0.11** | 0.03 | -0.01 | -0.10 |
| ***Slide*** | -0.03 | 0.07 | **0.35** | -0.04 |  | **-0.10** | **-0.38** | **0.32** |
| ***Rise*** | -0.05 | **0.19** | **-0.17** | -0.09 | -0.13 |  | 0.02 | **-0.41** |
| ***Prop.av*** | -0.13 | **-0.22** | **-0.26** | **-0.15** | **-0.38** | **0.16** |  | **0.11** |
| ***Cup*** | -0.05 | **-0.53** | -0.11 | 0.01 | **0.31** | **-0.45** | 0.10 |  |
| ***BDNA***  ***ADNA*** | ***Tilt*** | ***Roll*** | ***Twist*** | ***Shift*** | ***Slide*** | ***Rise*** | ***Prop.av*** | ***Cup*** |
| ***Tilt*** |  | 0.03 | 0.01 | **0.44** | 0.01 | 0.13 | 0.05 | -0.08 |
| ***Roll*** | -0.04 |  | **-0.67** | 0.12 | -0.05 | 0.13 | **0.25** | 0.23 |
| ***Twist*** | -0.16 | -0.24 |  | -0.18 | **0.31** | -0.09 | -0.14 | **-0.52** |
| ***Shift*** | **0.64** | 0.04 | -0.27 |  | -0.09 | 0.01 | 0.02 | 0.05 |
| ***Slide*** | -0.11 | 0.00 | **0.46** | -0.11 |  | **0.39** | **0.40** | 0.15 |
| ***Rise*** | -0.18 | 0.13 | -0.19 | -0.25 | -0.31 |  | **0.30** | -0.13 |
| ***Prop.av*** | 0.07 | -0.11 | -0.25 | 0.11 | -0.18 | 0.17 |  | **0.27** |
| ***Cup*** | 0.12 | -0.29 | -0.07 | 0.09 | **0.35** | **-0.55** | 0.05 |  |
