# Supplementary material for: Sequence dependent variations in RNA duplex are related to non-canonical hydrogen bond interactions in dinucleotide steps
Source: BMC Res Notes. 2014 Feb 7;7:83. doi: 10.1186/1756-0500-7-83 (PMC3930292; doi:10.1186/1756-0500-7-83)
Supplement: Additional file 4 — Partial charges assigned to atoms in Adenine and Guanine nucleosides. The partial charges derived from Natural Bond Orbital (NBO) and Electrostatic potential (ESP) calculations are listed, along with those used in AMBER and CHARMM force fields. The large negative charge assigned by NBO calculation is highlighted in bold for the N9 atoms of adenine and guanine bases, which are involved in an unusual cross-strand hydrogen bond with the 2-amino group of guanine in the CA/UG and CG/CG steps respectively (shown in Figure 7a and b). [file 1756-0500-7-83-S4.doc]

**Additional file 4 – Partial charges assigned to atoms in Adenine and Guanine nucleosides**

The partial charges derived from Natural Bond Orbital (NBO) and Electrostatic potential (ESP) calculations are listed, along with those used in AMBER and CHARMM force fields. The large negative charge assigned by NBO calculation is highlighted in bold for the N9 atoms of adenine and guanine bases, which are involved in an unusual cross-strand hydrogen bond with the 2-amino group of guanine in the CA/UG and CG/CG steps respectively (shown in **Figures 7a** and **7b**). (DOC

**format)**

| ADE | | | | | GUA | | | | |
| --- | --- | --- | --- | --- | --- | --- | --- | --- | --- |
| **ATOM** | **NBO** | **ESP** | **CHARMM** | **AMBER** | **ATOM** | **NBO** | **ESP** | **CHARMM** | **AMBER** |
| **Base** | | | | | **Base** | | | | |
| N1 | -0.7022 | -0.7918 | -0.74 | -0.7615 | N1 | -0.7664 | -0.8393 | -0.34 | -0.4787 |
| C2 | 0.4411 | 0.6270 | 0.50 | 0.5875 | C2 | 0.8504 | 0.9176 | 0.75 | 0.7657 |
|  |  |  |  |  | N2 | -0.8956 | -0.8424 | -0.68 | -0.9672 |
| N3 | -0.6738 | -0.7633 | -0.75 | -0.6997 | N3 | -0.7454 | -0.7525 | -0.74 | -0.6323 |
| C4 | 0.5180 | 0.3723 | 0.43 | 0.3053 | C4 | 0.4888 | 0.3190 | 0.26 | 0.1222 |
| C5 | -0.0402 | 0.0249 | 0.28 | 0.0515 | C5 | -0.0905 | -0.0127 | 0.00 | 0.1744 |
| C6 | 0.5894 | 0.6943 | 0.46 | 0.7009 | C6 | 0.8589 | 0.7671 | 0.54 | 0.4770 |
| N6 | -0.8821 | -0.8334 | -0.77 | -0.9019 | O6 | -0.7268 | -0.6017 | -0.51 | -0.5597 |
| N7 | -0.5815 | -0.5825 | -0.71 | -0.6073 | N7 | -0.5369 | -0.5346 | -0.60 | -0.5709 |
| C8 | 0.3755 | 0.1202 | 0.34 | 0.2006 | C8 | 0.3374 | 0.0957 | 0.25 | 0.1374 |
| **N9** | **-0.5768** | **-0.0099** | **-0.05** | **-0.0251** | **N9** | **-0.5816** | **-0.0529** | **-0.02** | **0.0492** |
|  |  |  |  |  |  |  |  |  |  |
| **Sugar** | | | | | **Sugar** | | | | |
| O5* | -0.8239 | -0.7051 | -0.66 | -0.6223 | O5* | -0.8078 | -0.6764 | -0.66 | -0.6223 |
| C5* | 0.0067 | 0.0315 | 0.05 | 0.0558 | C5* | 0.0028 | 0.2700 | 0.05 | 0.0558 |
| C4* | 0.1246 | 0.0624 | 0.16 | 0.1065 | C4* | 0.1268 | 0.1187 | 0.16 | 0.1065 |
| O4* | -0.6960 | -0.4567 | -0.50 | -0.3548 | O4* | -0.6829 | -0.4570 | -0.50 | -0.3548 |
| C3* | 0.1210 | 0.2839 | 0.14 | 0.2022 | C3* | 0.1251 | 0.6496 | 0.14 | 0.2022 |
| O3* | -0.8143 | -0.6831 | -0.66 | -0.6541 | O3* | -0.8050 | -0.7319 | -0.66 | -0.6541 |
| C2* | 0.1231 | 0.1394 | 0.14 | 0.0670 | C2* | 0.1202 | -0.1391 | 0.14 | 0.0670 |
| O2* | -0.8015 | -0.6636 | -0.66 | -0.6139 | O2* | -0.8269 | -0.6094 | -0.66 | -0.6139 |
| C1* | 0.3643 | 0.3571 | 0.16 | 0.0394 | C1* | 0.3902 | 0.3069 | 0.16 | 0.0191 |
|  |  |  |  |  |  |  |  |  |  |
| **Hydrogen** | | | | | **Hydrogen** | | | | |
| H5* | 0.1693 | 0.0521 | 0.09 | 0.0679 | H5* | 0.1934 | 0.0621 | 0.09 | 0.0679 |
| H5** | 0.1822 | 0.1065 | 0.09 | 0.0679 | H5** | 0.1773 | -0.0338 | 0.09 | 0.0679 |
| H4* | 0.2026 | 0.0796 | 0.09 | 0.1174 | H4* | 0.1930 | 0.0165 | 0.09 | 0.1174 |
| H3* | 0.2123 | 0.1027 | 0.09 | 0.0615 | H3* | 0.1954 | -0.0613 | 0.09 | 0.0615 |
| H3T | 0.4871 | 0.4145 | 0.43 | 0.4376 | H3T | 0.5085 | 0.4181 | 0.43 | 0.4376 |
| H2* | 0.2131 | 0.0948 | 0.09 | 0.0972 | H2* | 0.2013 | 0.0894 | 0.09 | 0.0972 |
| H2** | 0.5055 | 0.4229 | 0.43 | 0.4186 | H2** | 0.5001 | 0.4219 | 0.43 | 0.4186 |
| H1* | 0.2144 | 0.0436 | 0.09 | 0.2007 | H1* | 0.2085 | 0.0987 | 0.09 | 0.2006 |
| H8 | 0.2169 | 0.1874 | 0.12 | 0.1553 | H8 | 0.2346 | 0.2088 | 0.16 | 0.1640 |
| H61 | 0.4210 | 0.3867 | 0.38 | 0.4115 | H21 | 0.4079 | 0.3560 | 0.32 | 0.4364 |
| H62 | 0.4235 | 0.3730 | 0.38 | 0.4115 | H22 | 0.4214 | 0.3757 | 0.35 | 0.4364 |
| H2 | 0.1855 | 0.0539 | 0.13 | 0.0473 | H1 | 0.4384 | 0.4335 | 0.26 | 0.3424 |
| H5T | 0.4954 | 0.4587 | 0.43 | 0.4295 | H5T | 0.4853 | 0.4197 | 0.43 | 0.4295 |
